# Acetylation-Dependent Regulation of Notch Signaling in Macrophages by SIRT1 Affects Sepsis Development

**DOI:** 10.3389/fimmu.2018.00762

**Published:** 2018-05-07

**Authors:** Xiaozhi Bai, Ting He, Yang Liu, Julei Zhang, Xiaoqiang Li, Jihong Shi, Kejia Wang, Fu Han, Wei Zhang, Yijie Zhang, Weixia Cai, Dahai Hu

**Affiliations:** Department of Burns and Cutaneous Surgery, Xijing Hospital, Fourth Military Medical University, Xi’an, China

**Keywords:** sepsis, SIRT1, Notch, macrophage, deacetylate

## Abstract

SIRT1 is reported to participate in macrophage differentiation and affect sepsis, and Notch signaling is widely reported to influence inflammation and macrophage activation. However, the specific mechanisms through which SIRT1 regulates sepsis and the relationship between SIRT1 and Notch signaling remain poorly elucidated. In this study, we found that SIRT1 levels were decreased in sepsis both *in vitro* and *in vivo* and that SIRT1 regulation of Notch signaling affected inflammation. In lipopolysaccharide (LPS)-induced sepsis, the levels of Notch signaling molecules, including Notch1, Notch2, Hes1, and intracellular domain of Notch (NICD), were increased. However, NICD could be deacetylated by SIRT1, and this led to the suppression of Notch signaling. Notably, in macrophages from myeloid-specific *RBP-J*^−/−^ mice, in which Notch signaling is inhibited, pro-inflammatory cytokines were expressed at lower levels than in macrophages from wild-type littermates and in *RBP-J*^−/−^ macrophages, and the NF-κB pathway was also inhibited. Accordingly, in the case of *RBP-J*^−/−^ mice, LPS-induced inflammation and mortality were lower than in wild-type mice. Our results indicate that SIRT1 inhibits Notch signaling through NICD deacetylation and thus ultimately alleviates sepsis.

## Introduction

Sepsis is a life-threatening condition caused by uncontrolled immune response triggered by infections (1, 2). Although our understanding of sepsis has increased substantially in recent years, sepsis is still reported to be the leading cause of death in seriously ill patients, and the incidence of sepsis has been increasing every year (3, 4). In both the initiation and the resolution of immune responses, macrophages function as crucial coordinators (5), and the polarization of macrophages, which are involved in the process of sepsis, is influenced by the microenvironment. Accordingly, macrophages can be roughly divided into M1 and M2 macrophages: M1 macrophages are activated by lipopolysaccharide (LPS) and inflammatory cytokines such as interferon γ (6), and, once activated, M1 macrophages release various pro-inflammatory cytokines, including TNF-α, IL-1β, IL-12, and IL-23 (7–9). However, macrophages can also be activated by IL-4 or IL-10 and polarize into M2 macrophages, which are associated with the secretion of anti-inflammatory cytokines (10). For investigating the mechanism of sepsis, the LPS-induced inflammation model is extensively used (11, 12).

SIRT1, a highly conserved mammalian NAD^+^-dependent histone deacetylase, is reported to be associated with protection against inflammation (13). SIRT1 inhibits the production of pro-inflammatory cytokines in macrophages (14), and our group has reported that SIRT1 alleviates LPS-induced sepsis (15). Intriguingly, recent studies have shown that SIRT1negatively affects the Notch signaling in several contexts.

Over the last decade, Notch signaling has been shown to regulate multiple cell fate decision and cell differentiation processes during development and to function within the immune system. Mammals express four Notch receptors (Notch1–4) and five ligands (Delta-like 1, 3, and 4 and Jagged 1 and 2). During the activation of Notch signaling, the intracellular domain of Notch (NICD) traffics to the nucleus and heterodimerizes with the DNA-binding transcription factor CSL to form a nuclear transcription complex and induce the transcriptional expression of downstream target genes (16). In mouse, the CSL is known as RBP-J. Studies conducted using genome-wide expression analyses and chromatin immunoprecipitation (IP) arrays have revealed that numerous genes can be directly regulated by Notch (17, 18), and members of the Hairy enhancer of split (Hes) or Hairy related (Hey or Hrt) gene family have been identified as Notch target genes in several tissues. Canonical Notch signaling was recently shown to participate in macrophage activation and death (19), and the canonical *Notch-RBP-J* pathway was reported to contribute to TLR-induced cytokine gene expression during macrophage activation in inflammation (20–23). Moreover, inhibition of Notch signaling was reported to alleviate hepatic ischemia–reperfusion injury (24). Conversely, increased Notch signaling in macrophages was found to be associated with atherosclerotic plaque formation through increased inflammation (25). The results of these studies demonstrated that the activation of Notch signaling contributes to the aggravation of inflammation.

SIRT1 has been reported to inhibit Notch-mediated transcription. In adult neural stem cells, SIRT1 was found to function as a key metabolic sensor for regulating adult hippocampal neuro-genesis, partly through its suppression of Notch signaling (26), and in chronic renal injury, endothelial SIRT1 was shown to counteract peritubular capillary rarefaction by repressing Notch1 signaling and antagonizing fibrosis (27). Moreover, SIRT1 and LSD1 were reported to interact directly and thereby affect histone deacetylation and repress the genes regulated by the Notch signaling pathway (28), and in Ewing sarcoma, Notch signaling was found to be abrogated, and the restoration of Notch signaling by using SIRT1 inhibitors caused tumor-growth arrest (29). However, SIRT1 has also been reported to positively regulate the Notch pathway in *Drosophila*, and this might be context-dependent (30). Based on these findings and on our previous studies, we investigated the contribution of Notch in sepsis and the association of SIRT1 with Notch signaling.

## Materials and Methods

### Animals

This study was conducted in accordance with the principles of ARRIVE (31) approved by the Ethics Committee of Xijing Hospital, affiliated with the Fourth Military Medical University (No: XJYYLL-2015206). Eight-week-old SPF healthy male C57BL/6 mice, myeloid-specific *RBP-J* knockout mice (*RBP-J*^−/−^mice) (32), myeloid-specific *sirt1* knockout mice (*sirt1*^−/−^mice) on C57BL/6 background, and littermate wild-type (WT) mice, weighing 20–25 g, were purchased from the Experimental Animal Center of The Fourth Military Medical University. Briefly, to generate myeloid-specific *sirt1*^−/−^ mice, mice carrying a floxed *sirt1* allele (*Sirt1*^flox/flox^; Jackson Laboratory), in which exon 4 of *sirt1* was flanked by loxP sites, were crossed with *Lyz2-Cre* transgenic mice (Jackson Laboratory) (33). Similarly, to generate myeloid-specific *RBP-J*^−/−^ mice, mice carrying the *Lyz2-Cre* transgene were crossed with *RBP-J-floxed* mice (*RBP-J*^flox/flox^ Jackson Laboratory) (34). Mice were genotyped by performing PCR on tail-derived DNA. All mice were housed under pathogen-free conditions and provided access to standard mouse food and water *ad libitum*. LPS solution was purchased from Sigma (St. Louis, MO, USA) and diluted with PBS, and the animal model of sepsis was generated by injecting mice intraperitoneally with LPS (10 mg/kg bodyweight). In certain experiments, C57BL/6 mice were injected intravenously with either PBS or the SIRT1 activator SRT1720 (Selleckchem, Houston, TX, USA; 20 mg/kg bodyweight). We separately injected 20 *RBP-J*^−/−^mice and WT mice and then closely monitored them for 72 h after LPS injection and calculated the survival rate. Subsequently, we randomly selected six each of the gene-knockout mice and their WT littermates, injected the mice with LPS, sacrificed them 24 h later, and then collected blood from left ventricle, peritoneal macrophages, and heart, liver, lung, and kidney tissues. Mice were anesthetized using 1% sodium pentobarbital (0.5 ml/100 g bodyweight), and all procedures were performed in a clean surgical room, using sterilized instruments. Every effort was made to minimize the suffering of the mice.

### Cell Culture

Cells of the murine macrophage line RAW264.7 (ATCC, USA) were cultured in RPMI 1640 medium (Gibco, USA) containing 10% fetal bovine serum (Excell Bio, China) in a humidified 5% CO_2_, 95% air atmosphere. Peritoneal macrophages from mice were collected and incubated as described in other literature (35). The medium was changed every 48 h, and all experiments were performed using cells between the third and fifth passages.

### Cell Grouping and Treatment

RAW264.7 cells were seeded in six-well plates, grown to 60–80% confluence, serum-starved for 12 h, and divided into four groups: control, SRT1720, LPS, and SRT1720 + LPS. Cells in the SRT1720, LPS, and SRT1720 + LPS groups were exposed for 4 h to, respectively, 100 nM SRT1720, 1 µg/ml LPS, and both SRT1720 and LPS. Control group cells were maintained for 4 h in medium containing an equivalent amount of PBS as SRT1720/LPS.

SIRT1-specific siRNA and scrambled siRNA were designed and constructed by Shanghai Gene Pharma Co., Ltd., China. The siRNAs were transfected into RAW264.7 macrophages, according to the instructions provided with Lipofectamine 2000 (Invitrogen Inc., CA, USA), and then these two types of cells (SIRT1-knockdown and control) were exposed for 4 h to 1 µg/ml LPS or the same volume of PBS.

In other experiments, we used two pairs of mouse peritoneal macrophages: *sirt1*^−/−^ macrophages and macrophages from littermate WT mice, and *RBP-J*^−/−^ macrophages and macrophages from littermate WT mice. These macrophages were seeded in six-well plates, grown to 60–80% confluence, serum-starved for 12 h, and then exposed for 4 h to either 1 µg/ml LPS or an equivalent volume of PBS.

### IP and Co-Immunoprecipitation (co-IP)

Briefly, we carefully washed macrophages twice with pre-chilled PBS and added in cold RIPA lysis buffer (1 ml/10^7^cells), and then scraped the cells into clean 1.5 ml Eppendorf tubes and agitated the tubes on a low-speed rotating shaker for 15 min at 4°C. After centrifugation at 14,000 × *g* (4°C, 15 min), the supernatant was immediately transferred into clean tubes. Protein A/G-agarose beads were washed twice with PBS and a 50% protein A/G agarose working solution was prepared (in PBS); this working solution was added at a ratio of 100 µl for 1 ml of sample solution, and the tubes were shaken on a horizontal shaker for 10 min at 4°C and then centrifuged at 14,000 × *g* (4°C, 15 min). Subsequently, the supernatant was transferred into new tubes, and the protein A/G-agarose beads were discarded. Next, the beads were added again to the samples, and after centrifugation at 14,000 × *g* (4°C, 15 min), the supernatant was transferred into clean tubes and the beads were discarded. Total protein concentration in cell extracts was measured using the BCA assay, and the protein concentration was lowered to 1 µg/µl by adding PBS to reduce the concentration of detergents.

For IP, we mixed cell extracts with antibodies against IgG (1:1,000, Abcam, Cambridge, UK), acetyl-lysine (1:300, Abcam; or 1:1,000, CST, USA), or NICD (1:200, Abcam), and for co-IP, we added anti-SIRT1 (1:1,000, Abcam); a total volume of 500 µl was used in both cases, and the samples were slowly shaken on a rotating shaker overnight at 4°C. After centrifugation at 14,000 × *g* for 5 s, the pellets were retained and washed thrice with pre-chilled washing buffer, and then the captured proteins were analyzed by means of Western blotting. The supernatants were also collected and used in Western blotting assays.

### Western Blotting

Total-protein samples (50 µg/lane) from tissues or macrophages were separated using SDS-PAGE and transferred to PVDF membranes, which were blocked with 5% non-fat milk at room temperature for 3 h and then incubated (4°C, overnight) with primary antibodies against SIRT1 (1:1,000, Abcam, Cambridge, UK), p65 (1:1,000, CST, USA), p-p65 (1:1,000, CST), IκB-α (1:1,000, CST, USA), p-IκB-α (1:1,000, CST, USA), NICD (1:500, Abcam, Cambridge, UK), GAPDH (1:1,000, CST, USA), or tubulin (1:1,000, CST, USA). Next, the membranes were incubated (37°C, 1 h) with HRP-conjugated secondary antibodies (1:3,000, Boster, Wuhan, China), and then the protein bands were developed using a developing solution. Results were analyzed using ImageJ 5.01 and normalized against β-actin.

### Total RNA Extraction and qRT-PCR

RNA was extracted using Trizol reagent (Invitrogen Inc., CA), according to the products’ instructions. And 500 ng of the isolated RNA was reverse-transcribed taken for cDNA preparation. The RNA was reversely transcribed using a high-capacity cDNA synthesis kit (TaKaRa, Japan). The obtained cDNA was amplified in real-time RT-PCR assays performed using SYBR premix Ex TaqII (TaKaRa, Japan) and specific primers (Table 1) and the following amplification protocol: 40 cycles of denaturation by heating at 95°C for 30 s, annealing at 60°C for 34 s, and extension at 60°C for 1 min. Relative fold changes were calculated using the 2^−ΔΔCT^method and normalized against GAPDH.

**Table 1:** Primer sequences used for real-time-PCR analysis.

mRNA	Forward primer	Reverse primer
IL-1β	5′-TCCTGTGTAATGAAAGACGGC-3′	5′-TGCTTGTGAGGTGCTGATGTA-3′
IL-6	5′-GGGACTGATGCTGGTGACAA-3′	5′-TCCACGATTTCCCAGAGAACA-3′
TNF-α	5′-GAACTGGCAGAAGAGGCACT-3′	5′-CATAGAACTGATGAGAGGGAGG-3′
CCL2	5′-GTTAACGCCCCACTCACCTG-3′	5′-CCCATTCCTTCTTGGGGTCA-3′
NOS2	5′-GTGGTGTTCTTTGCTTCCAT-3′	5′-AGTAGTTGCTCCTCTTCCAA-3′
SIRT1	5′-TATTCCACGGTGCTGAGGTA-3′	5′-CACTTTCATCTTCCAAGGGTTC-3′
Notch1	5′-TGACAACTCCTACCTCTGCTTATG-3′	5′-GGTTCACAGGCACATTCGTA-3′
Notch2	5′-GTGTGACATTCCAGGACGCT-3′	5′-AGTGAAGTCGCCAGTCTGAC-3′
Hes1	5′-GGTCTACACCAGCAACAGTG-3′	5′-GGGCTAGGGACTTTACGGGT-3′
GAPDH	5′-GTGTTCCTACCCCCAATGTG-3′	5′-CATCGAAGGTGGAAGAGTGG-3′

### Hematoxylin and Eosin (H&E) Staining

Tissue specimens were acquired and fixed in 10% formalin, dehydrated in alcohol, embedded in paraffin, cut into 5 µm-thick sections, and then deparaffinized and stained with H&E. Pathological sections were examined and photographed under a microscope (All-in-one FSX100, Olympus, Japan), and for each section, six high-magnification images were randomly selected for blinded observation.

### Liver and Renal Function Evaluation

Creatinine (Cr) and blood urea nitrogen (BUN) are renal function indicators (36), and alanine aminotransferase (ALT) and aspartate transaminase (AST) are liver function indicators (37). Mice blood samples were collected to measure the serum levels of Cr, BUN, ALT, and AST by using a microplate reader (Infinite 200 PRO, Tecan, Switzerland).

### Plasmids and Transfection

SIRT1 H363Y is defective in deacetylase activity (38). The plasmid pBK/CMV-SIRT1-H363Y, which expresses the SIRT1 mutant harboring the histidine-to-tyrosine substitution at position 363, was purchased from Add gene (Cambridge, MA, USA), as was the WT*sirt1* plasmid. RAW264.7 cells were transfected with 0.25 µg of pBK/CMV-SIRT1 (WT or H363Y mutant), and the medium was changed at 24 h after transfection.

### Determination of Serum IL-1β, IL-6, and TNF-α Levels

The serum levels of TNF-α, IL-1β, and IL-6 were determined using commercial ELISA kits, according to the manufacturer’s instructions (Cusabio Biotech, Wuhan, China).

### Statistical Analysis

Data are presented as mean ± SD. Statistical differences between two groups were determined using Mann–Whitney *U* tests; for comparisons among multiple groups, one-way analysis of variance (ANOVA) was used. SPSS 18.0 program (IBM, Armonk, USA) was used for analyses, and *p* < 0.05 was considered statistically significant.

## Results

### LPS Stimulation Led to an Increase in the Levels of Pro-Inflammatory Cytokines in Macrophages and a Decrease in the Level of SIRT1

**Figure 1:**
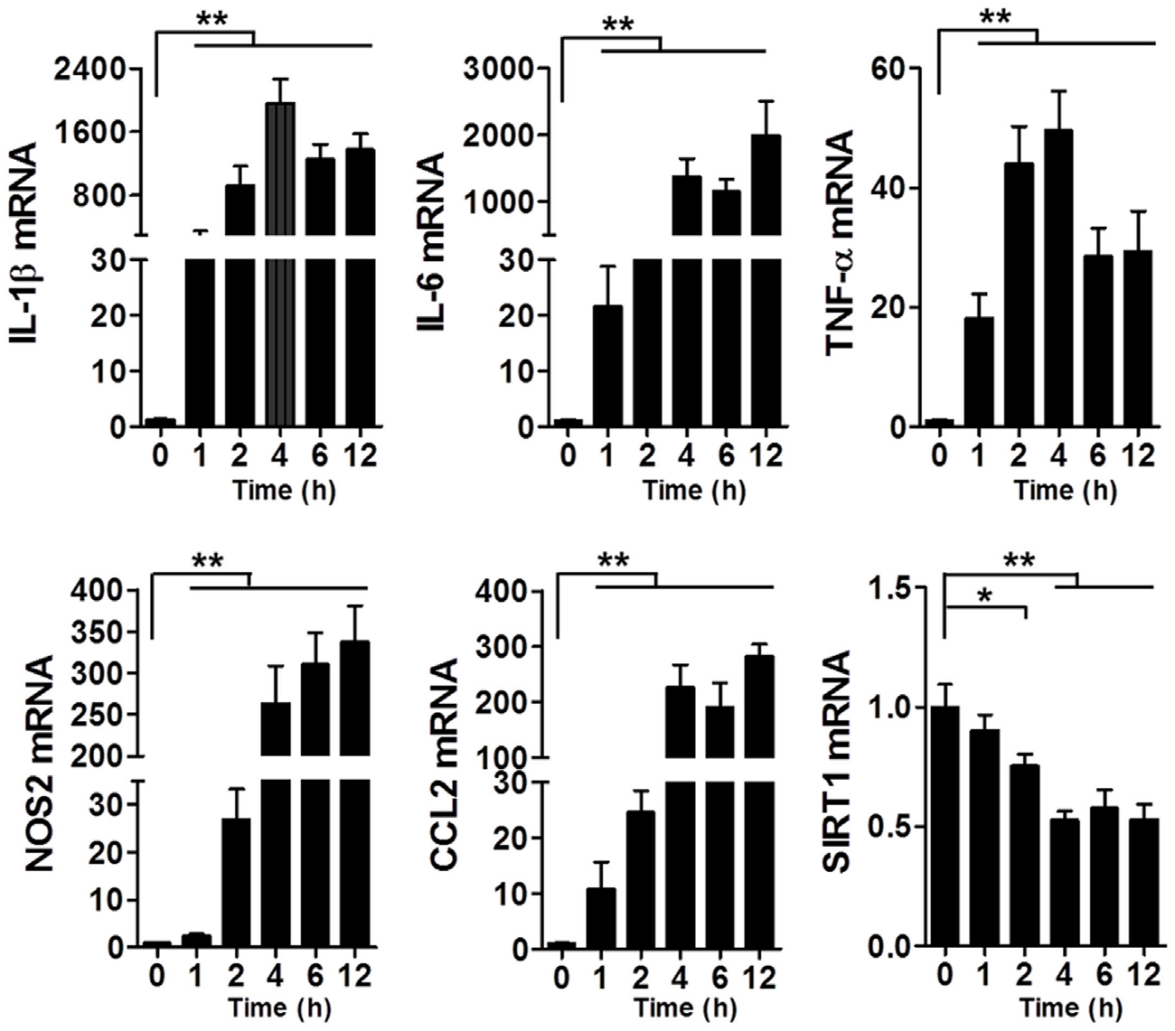
Levels of IL-1β, IL-6, TNF-α, NOS2, CCL2, and SIRT1 mRNAs in lipopolysaccharide-treated and untreated RAW264.7 cells. *p < 0.05, **p < 0.01; *n* = 6.

To assess the changes in SIRT1 levels in sepsis, we used the LPS-induced macrophage model (Figure 1): at 1–2 h after LPS stimulation, the mRNA levels of IL-1β, IL-6, TNF-α, NOS2, and CCL2 in RAW264.7 cells were significantly increased, which indicated that the macrophages were in a state of inflammation, whereas the SIRT1 mRNA level was decreased significantly.

### Levels of Pro-inflammatory Cytokines and SIRT1 in Macrophages Were Negatively Correlated

**Figure 2:**
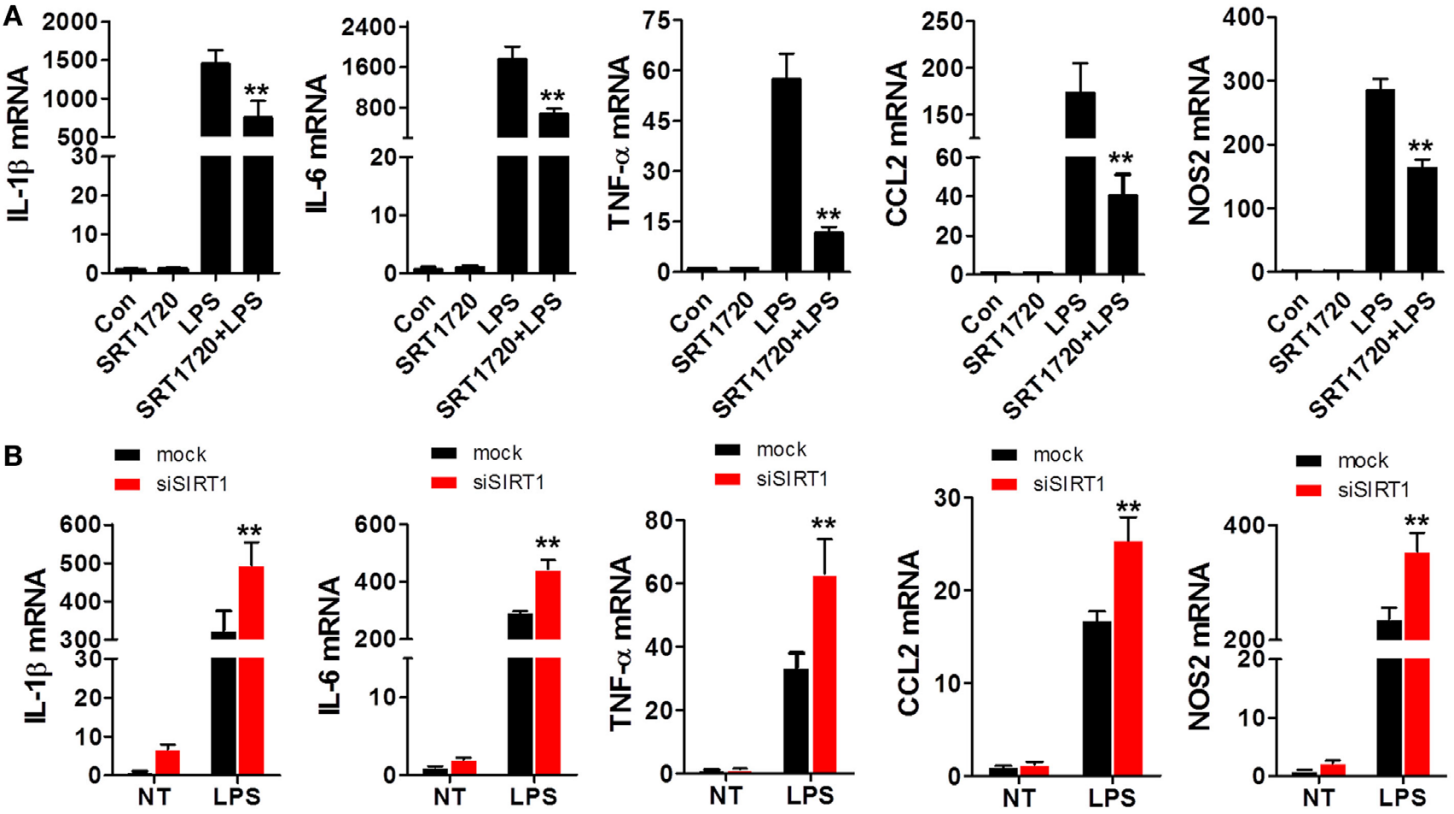
SIRT1 activation and inhibition led, respectively, to a decrease and increase in pro-inflammatory cytokines in macrophages. **(A)** RAW264.7 cells were treated with PBS (control) or SRT1720 or with lipopolysaccharide (LPS) or LPS + SRT1720, and then RT-PCR was performed to assess the expression levels of IL-1β, IL-6, TNF-α, NOS2, and CCL2. **p < 0.01 compared with the LPS group; *n* = 6. **(B)** RAW264.7 cells were transfected with SIRT1 siRNA or mock siRNA and then the knockdown and control cells were treated with LPS or PBS (NT). The expression levels of IL-1β, IL-6, TNF-α, NOS2, and CCL2 were examined using RT-PCR. **p < 0.01 compared with the LPS + mock group; *n* = 6.

Because SIRT1 levels were altered during sepsis as mentioned earlier, we sought to determine whether changes in SIRT1 levels affect the expression of pro-inflammatory cytokines. When macrophages were treated with both LPS and SRT1720, an activator of SIRT1 (39), the activation of SIRT1 was accompanied with a downregulation of pro-inflammatory cytokines as compared with their expression in cells treated with LPS alone (Figure 2A). Conversely, in macrophages transfected with SIRT1 siRNA, the levels of pro-inflammatory cytokines were higher than those in cells transfected with scrambled control siRNA (Figure 2B). Notably, in the case of mice injected with both LPS and SRT1720 (SRT1720 group), the survival rate at 72 h was significantly higher than that of mice injected with LPS only (Sham group), and, accordingly, pro-inflammatory cytokines were decreased in LPS + SRT1720 mice (Figure S1 in Supplementary Material). Furthermore, tissue H&E staining revealed more severe organ injury after LPS injection as compared with that after injection of both LPS and SRT1720 (Figure S2A in Supplementary Material), and kidney and liver function indicators were also increased in the sham-group mice (Figure S2B in Supplementary Material).

### Myeloid-Specific Knockout of *sirt1* Substantially Exacerbated LPS-Induced Organ Injury and Inflammation

**Figure 3:**
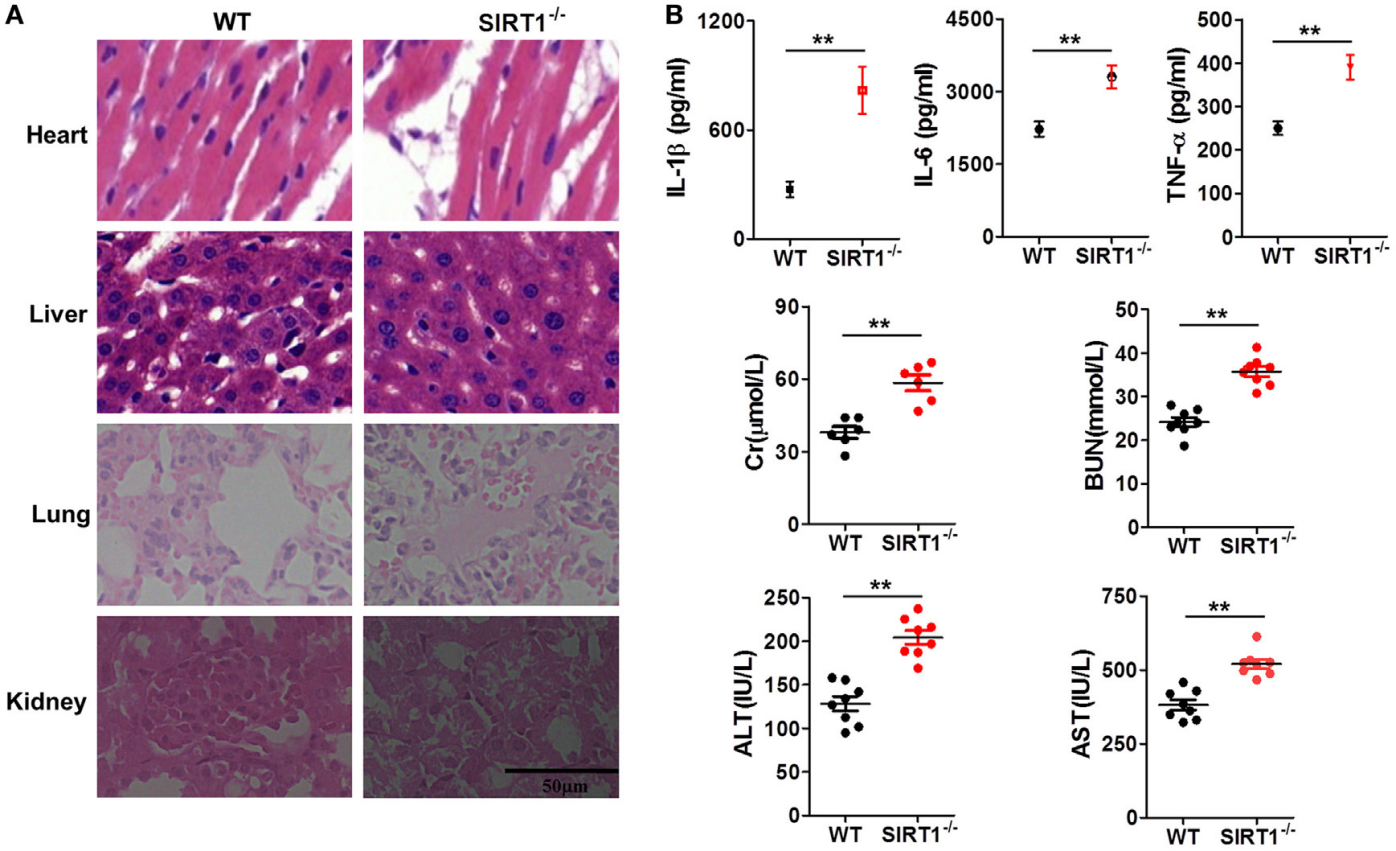
Lipopolysaccharide (LPS)-induced inflammation and organ injury were more severe in *sirt1*^−/−^ mice than in wild-type (WT) mice. **(A)** Hematoxylin and eosin (H&E) staining of heart, liver, lung, and kidney tissues from *sirt1*^−/−^ and WT mice after peritoneal injection of LPS. Sections were examined and photographed under a microscope. **(B)** Blood was collected from the left ventricle and serum IL-1β, IL-6, and TNF-α levels were examined and serum creatinine (Cr), blood urea nitrogen (BUN), alanine aminotransferase (ALT), and aspartate transaminase (AST) levels were measured using commercial ELISA kits.**p < 0.01 compared with WT mice; *n* = 6. Scale bar = 50 µm.

To further verify the relationship between SIRT1 and inflammation *in vivo*, myeloid-specific *sirt1*^−/−^ mice and littermate WT mice were intraperitoneally injected with LPS (10 mg/kg bodyweight). H&E staining (Figure 3A) revealed that as compared with the myocardium in WT mice, the myocardium in *sirt1*^−/−^ mice showed greater disorder in myocardial fiber arrangement, and some of the nuclei in myocardial cells were disrupted. In liver sections, there was significant considerable congestion of veins as well as hepatocyte necrosis in the case of *sirt1*^−/−^ mice. In pulmonary sections, drastic destruction of alveolar structures was detected in *sirt1*^−/−^ mice, and in these mice, the effusion in alveoli was markedly more severe than that in WT mice; moreover, tissue infiltration by inflammatory cells was substantially higher in *sirt1*^−/−^ mice than in WT mice. In the kidney, necrotic glomeruli were considerably more numerous in *sirt1*^−/−^ mice than in WT mice, and whereas the tubule structure was almost normal in WT mice, casts were observed in the tubules in *sirt1*^−/−^ mice. Finally, serum levels of IL-1β, IL-6, and TNF-α, as well as those of Cr, BUN, ALT, and AST, in *sirt1*^−/−^ mice were significantly higher than the corresponding levels in WT mice (Figure 3B).

### *Sirt1*^−/−^ Macrophages Expressed Markedly Higher Levels of Pro-Inflammatory Cytokines Than WT Macrophages After LPS Stimulation

**Figure 4:**
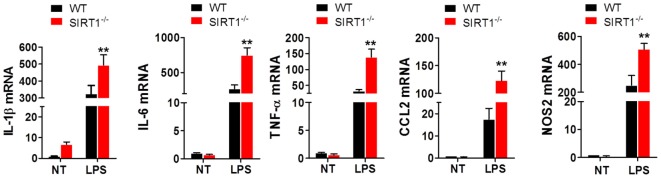
Lipopolysaccharide (LPS)-stimulated inflammation was exacerbated in *sirt1*^−/−^ macrophages. Wild-type (WT) and *sirt1*^−/−^ macrophages were treated with LPS or PBS (NT), and then RT-PCR was used to assess the levels of IL-1β, IL-6, TNF-α, CCL2, and NOS2. **p < 0.01 compared with the LPS + WT group; *n* = 6.

To clarify the effects of SIRT1 in sepsis, peritoneal macrophages were collected from *sirt1*^−/−^ and WT mice and treated with 1 µg/ml LPS for 4 h. The results of RT-PCR analyses showed that the mRNA levels of IL-1β, IL-6, TNF-α, CCL2, and NOS2 in *sirt1*^−/−^macrophages were considerably higher than those in WT macrophages (Figure 4).

### NICD Was Directly Associated with SIRT1 in LPS-Stimulated Macrophages

**Figure 5:**
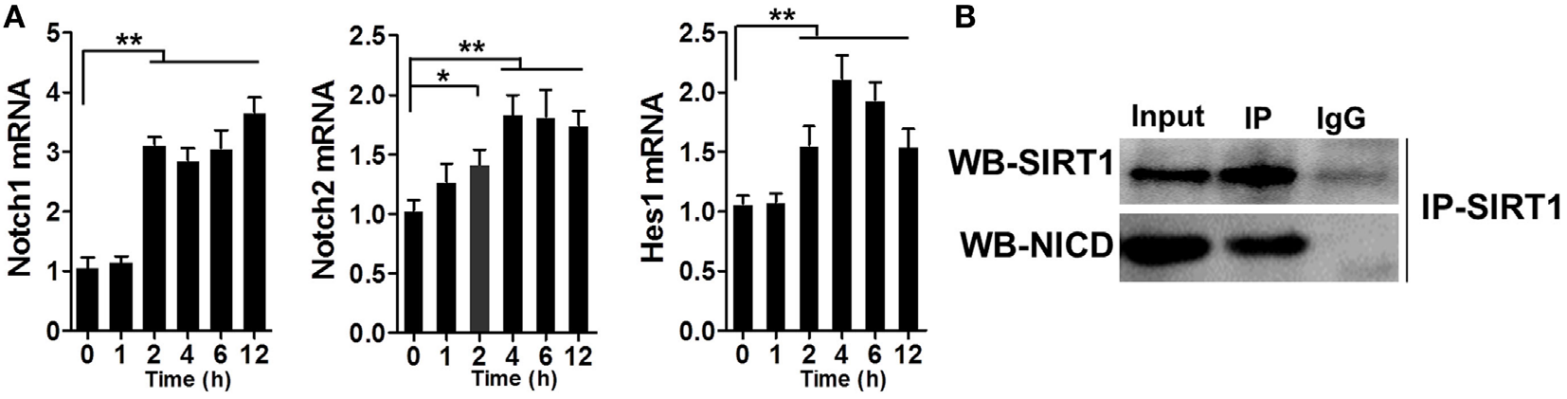
Direct interaction between Notch signaling and SIRT1 in macrophages. **(A)** RAW264.7 cells were treated with 1 µg/ml lipopolysaccharide (LPS), and then RT-PCR was used to examine the time-dependent expression of Notch1, Notch2, and Hes1. **p < 0.01 compared with 0 h; *n* = 6. **(B)** Co-immunoprecipitation of intracellular domain of Notch (NICD) and SIRT1. RAW264.7 cells were stimulated with LPS and then the cell extracts were used for immunoprecipitation (IP) with anti-NICD antibody and immunoblotting with anti-SIRT1 antibody, and for IP with anti-SIRT1 antibody and immunoblotting with anti-NICD antibody; *n* = 4.

Previous studies have reported the association of SIRT1 and Notch signaling. After verifying that SIRT1 was downregulated in LPS-induced sepsis, we tested whether Notch signaling was altered during this process. RAW264.7 cells were treated with 1 µg/ml LPS and then the mRNA levels of Notch1, Notch2, and Hes1 were examined at various time points, which revealed an increase in the mRNA levels within 2 h after treatment (Figure 5A). Furthermore, the results of co-IP experiments showed that NICD directly associated with SIRT1 in LPS-treated cells (Figure 5B).

### SIRT1 Inhibition in Macrophages Affected the Activation of Notch Signaling

**Figure 6:**
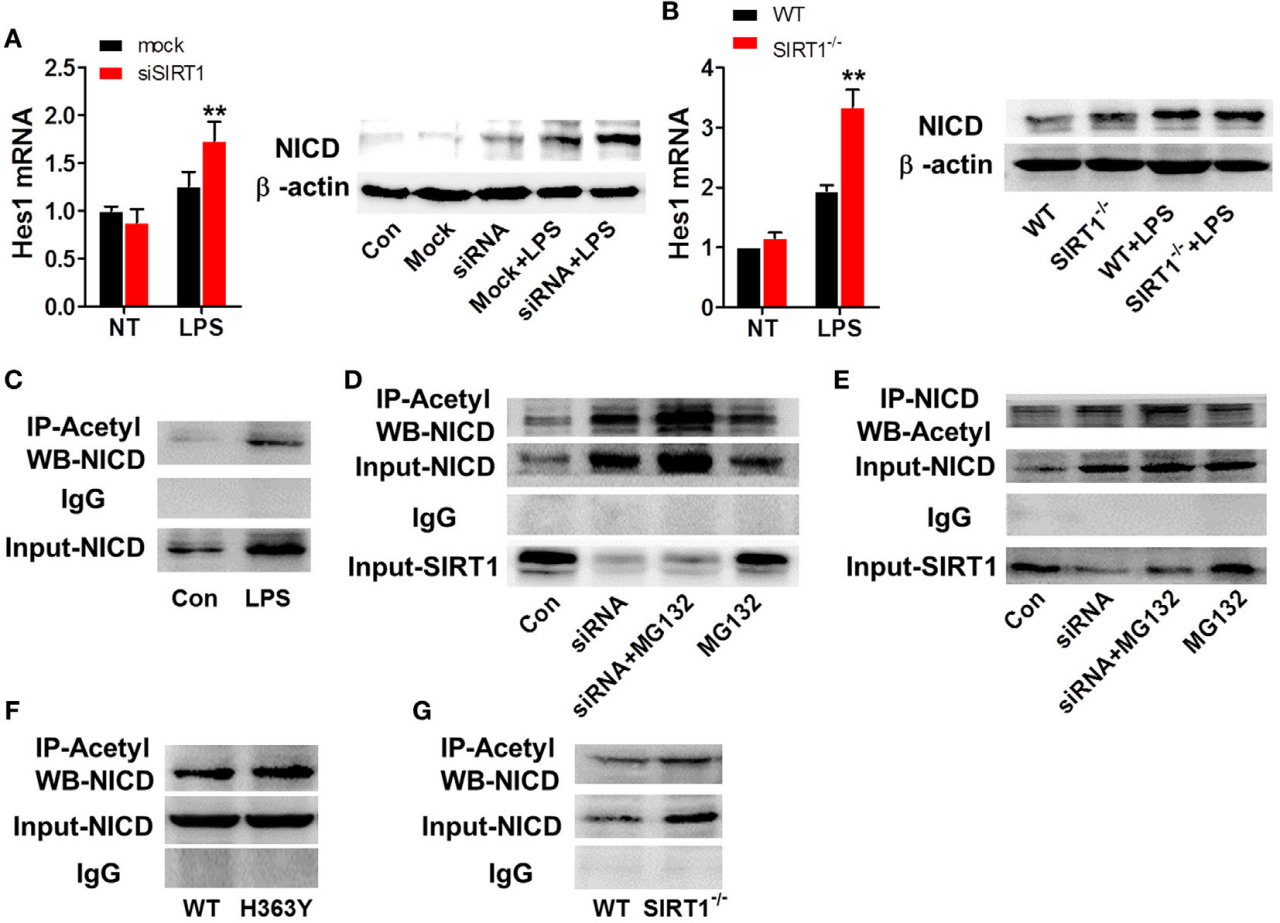
SIRT1 activation was negatively correlated with intracellular domain of Notch (NICD) levels. **(A)** RAW264.7 cells transfected with SIRT1 siRNA or mock siRNA were treated with lipopolysaccharide (LPS) or PBS, and then RT-PCR and Western blotting were used to assess Hes1 mRNA and NICD protein levels. **(B)** Wild-type (WT) and *sirt1*^−/−^ macrophages were treated with LPS or PBS and then Hes1 mRNA and NICD protein levels were examined using RT-PCR and Western blotting. **(C)** RAW264.7 cells were treated with LPS or PBS (control), and then the NICD acetylation level was evaluated by performing immunoprecipitation (IP) with anti-acetylation antibody and Western blotting with anti-NICD antibody. NICD expression in the whole-cell lysates was confirmed through Western blotting. **(D)** RAW264.7 cells were divided into control group (PBS), siSIRT1 group (SIRT1 siRNA + LPS), siSIRT1 + MG132 group (SIRT1 siRNA + LPS + MG132), and MG132 group (LPS + MG132), and NICD acetylation levels in the four groups were determined through IP with anti-acetylation antibody and Western blotting with anti-NICD antibody. SIRT1 expression in the whole-cell lysates was confirmed through Western blotting; *n* = 4. **(E)** Macrophages were separated into four groups and treated as in **(D)** and then NICD acetylation was determined by performing IP with anti-NICD antibody and Western blotting with anti-acetylation antibody. SIRT1 expression in the whole-cell lysates was confirmed through Western blotting; *n* = 3. **(F)** RAW264.7 cells were transfected with a plasmid expressing H363Y mutant SIRT1 or WT SIRT1, and then NICD acetylation was assessed through IP with anti-acetylation antibody and Western blotting with anti-NICD antibody. NICD expression in the whole-cell lysates was confirmed through Western blotting; *n* = 6. **(G)** WT and *sirt1*^−/−^ macrophages were stimulated with LPS and then NICD acetylation was examined by performing IP with anti-acetylation antibody and Western blotting with anti-NICD antibody. NICD expression in the whole-cell lysates was confirmed through Western blotting. **p < 0.01 compared with LPS + mock siRNA group; *n* = 3.

To further examine the relationship between SIRT1 and Notch signaling and the manner in which SIRT1 affects Notch signaling, we transfected RAW264.7 cells with SIRT1-specific siRNA and stimulated the cells with 1 µg/ml LPS; in these cells, the mRNA level of Hes1 and the protein level of NICD were higher than those in control cells treated with LPS (Figure 6A). Similarly, in macrophages from *sirt1*^−/−^mice, LPS treatment led to substantially higher upregulation of the Hes1 mRNA level and NICD protein level than in macrophages from WT mice (Figure 6B). Moreover, LPS treatment increased both the protein level and the acetylation of NICD (Figure 6C). Treatment with MG132, a proteasome inhibitor, can reduce the degradation of NICD, and following co-administration of MG132 and SIRT1 siRNA, the NICD level was substantially higher than that after treatment of cells with MG132 alone, and the level of acetylated NICD was also increased after transfection of cells with the SIRT1 siRNA (Figures 6D,E). Moreover, in RAW264.7 cells transfected with H363Y SIRT1 and stimulated with LPS, the level of acetylated NICD was higher than that in cells transfected with WT SIRT1 and treated with LPS (Figures 6F,G). These results suggest that the interaction of NICD and SIRT1 leads to the deacetylation and degradation of NICD.

### *RBP-J*^−/−^ Macrophages Showed Milder Inflammation Than WT Macrophages After LPS Stimulation

**Figure 7:**
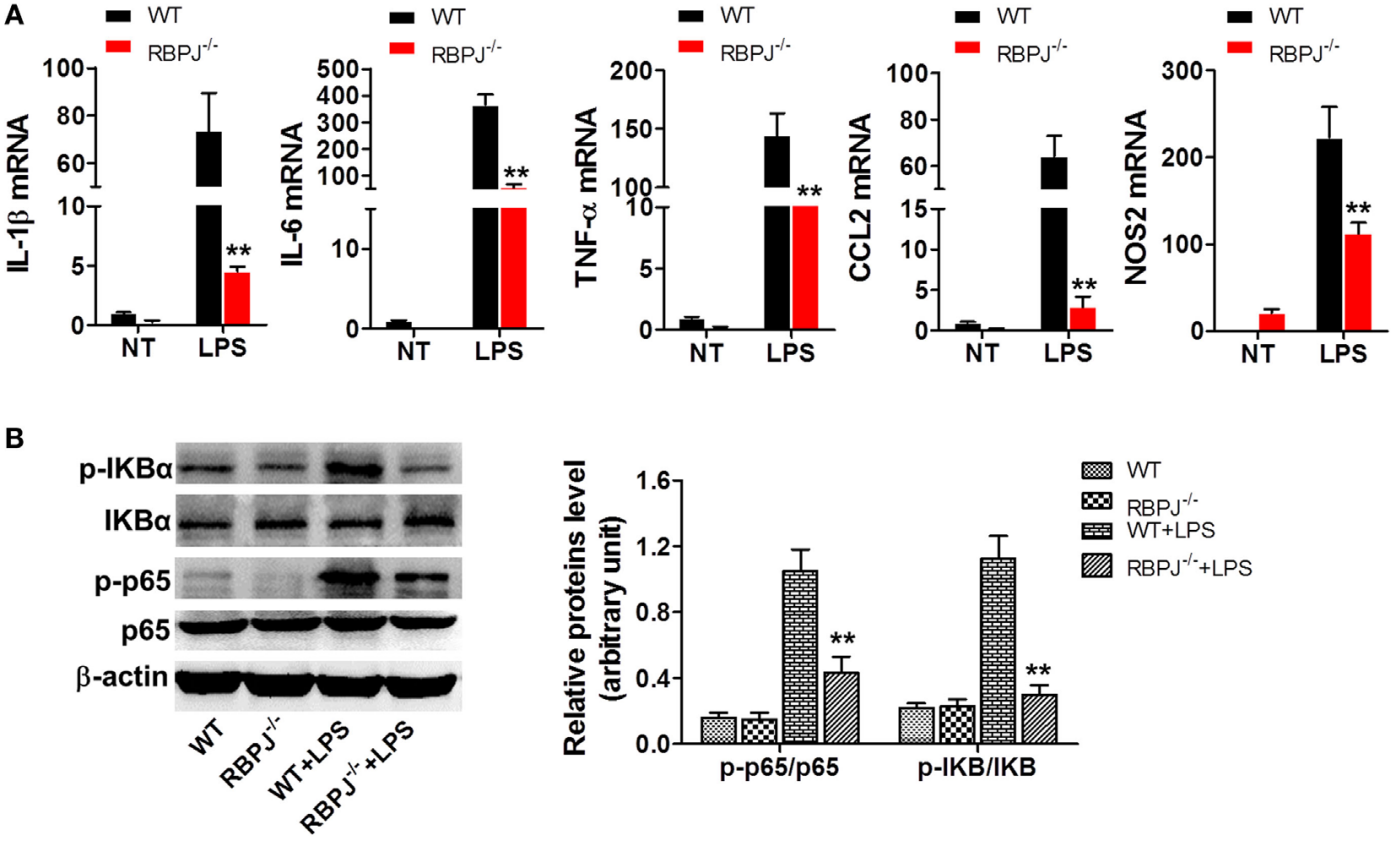
*RBP-J* knockout led to decreased inflammation in macrophages. **(A)** *RBP-J*^−/−^ and wild-type (WT) macrophages were stimulated with lipopolysaccharide (LPS) and then RT-PCR was used to assess the levels of IL-1β, IL-6, TNF-α, CCL2, and NOS2. **(B)** *RBP-J*^−/−^ and WT macrophages were treated with LPS or PBS and then the phosphorylation levels of p65 and IκBα were examined through Western blotting. **p < 0.01 compared with LPS + WT group; *n* = 6.

Given that SIRT1 was found to influence the activation of Notch signaling, we investigated whether the regulation of Notch signaling affects inflammation and the NF-κB pathway, which is closely associated with inflammation. Peritoneal macrophages were acquired from *RBP-J*^−/−^ and WT mice at 48 h after LPS injection, and measurement of mRNA levels revealed significantly higher expression of IL-1β, IL-6, TNF-α, CCL2, and NOS2 in WT macrophages than in *RBP-J*^−/−^ macrophages (Figure 7A). Moreover, after LPS stimulation, the phosphorylation of IκBα and p65 was markedly higher in WT mice than in *RBP-J*^−/−^ mice (Figure 7B).

### In Myeloid-Specific *RBP-J*^−/−^ mice, LPS-Induced Organ Injury and Inflammation Were Alleviated

**Figure 8:**
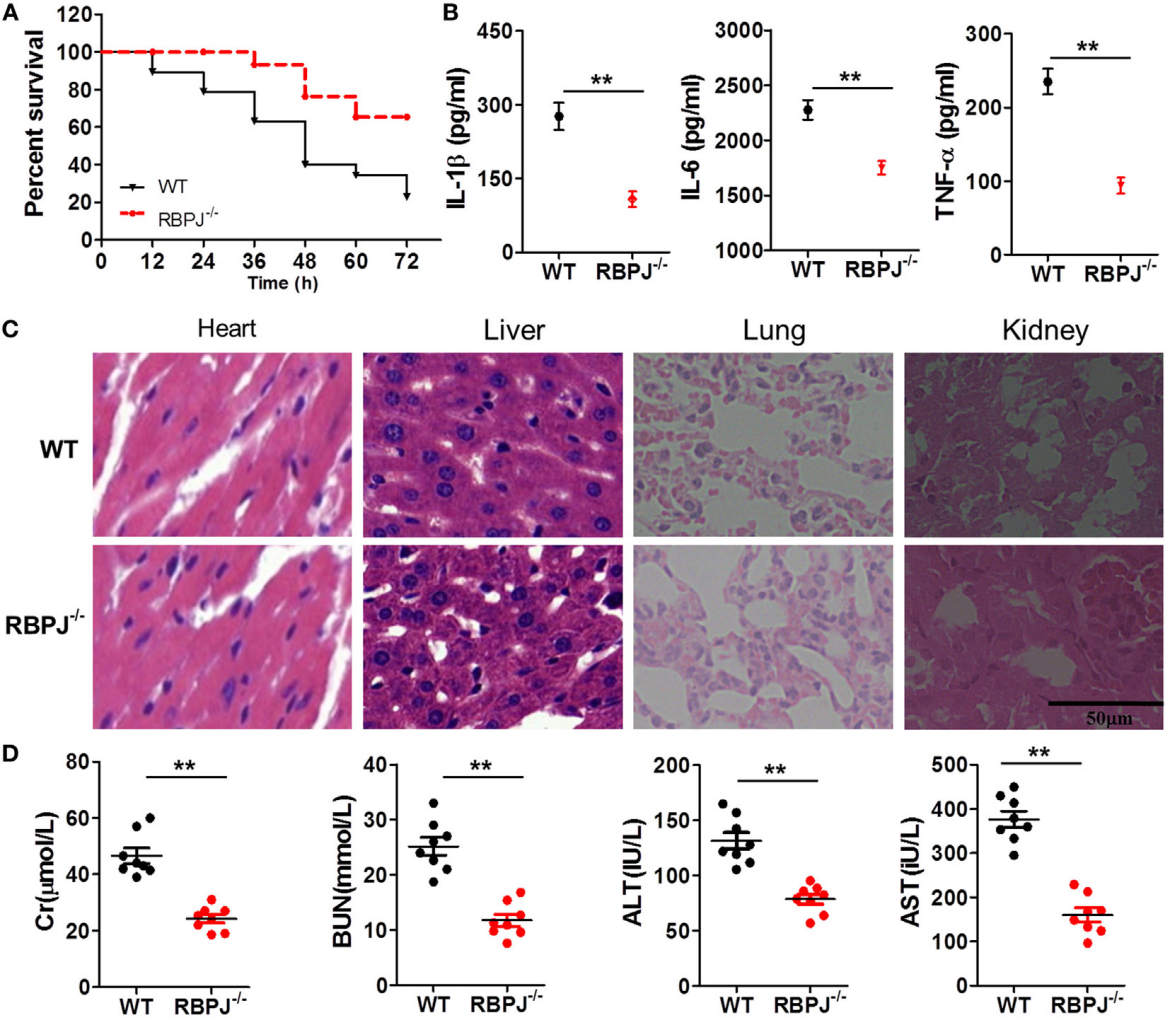
*RBP-J* knockout resulted in decreased inflammation *in vivo*. **(A)** Survival rates of wild-type (WT) and *RBP-J*^−/−^ mice after 72-h stimulation with lipopolysaccharide (LPS). **(B)** IL-1β, IL-6, and TNF-α levels in blood were measured using a microplate reader. **(C)** Hematoxylin and eosin staining of heart, liver, lung, and kidney tissues from *RBP-J*^−/−^ and WT mice after peritoneal injection of LPS. Sections were examined and photographed under a microscope. **(D)** Levels of creatinine (Cr), blood urea nitrogen (BUN), alanine aminotransferase (ALT), and aspartate transaminase (AST) in blood were determined using commercial ELISA kits. **p < 0.01 compared with WT mice; *n* = 6. Scale bar = 50 µm.

Finally, we examined the *in vivo* impact of Notch signaling on inflammation. After LPS injection, the mortality of *RBP-J*^−/−^ mice was significantly lower than that of WT mice (Figure 8A), and the serum levels of IL-1β, IL-6, and TNF-α in *RBP-J*^−/−^ mice were drastically lower than those in WT mice (Figure 8B). Moreover, H&E staining revealed that the effects of LPS were noticeably mitigated in *RBP-J*^−/−^ mice (Figure 8C): as compared with the myocardium in WT mice, the myocardium in *RBP-J*^−/−^ mice showed a more ordered arrangement of fibers, and the nuclei of myocardial cells were almost intact in the knockout mice; liver sections from WT mice showed congestion of veins and hepatocyte necrosis, but in the case of *RBP-J*^−/−^ mice, these pathological changes were milder. In pulmonary sections from WT mice, alveolar structures were found to be more drastically damaged; the effusion in alveoli was markedly more severe relative to what was observed in sections from *RBP-J*^−/−^ mice; and infiltration by inflammatory cells in pulmonary tissue was considerably more severe in WT mice than in *RBP-J*^−/−^ mice. In the kidney, substantially fewer necrotic glomeruli were detected in *RBP-J*^−/−^ mice than in WT mice. Although the tubule structure was almost normal in *RBP-J*^−/−^ mice, casts were clearly detected in the tubules in WT mice. Finally, the serum levels of Cr, BUN, AST, and ALT were lower in *RBP-J*^−/−^ mice than in WT mice (Figure 8D).

## Discussion

Notch signaling, which is highly conserved from *Drosophila* to mammals, plays a fundamental role during embryonic development that is associated with the control of cell proliferation/differentiation and apoptosis through cell interaction (40). Notch signaling is widely recognized to participate in myeloid-cell development and differentiation (41), and both Notch ligands and receptors are expressed on the surface of macrophages, which means that macrophages can not only induce but also respond to Notch signals (42). Recent evidence suggests that Notch signaling is closely associated with innate immunity and inflammation (42). In RAW264.7 cells, NICD overexpression increased COX2 levels in response to *Mycobacterium bovis* BCG infection, whereas transfection with Notch1 siRNA blocked the effect (43). Moreover, NICD1, Hes1, and MMP9 levels were increased in brain tissue samples from patients with tuberculosis meningitis (44). Furthermore, Notch signaling was reported to be activated during the early stage of septic shock and to participate in the regulation of PD-1 expression, and inhibition of Notch signaling suppressed PD-1 expression and alleviated sepsis (45). However, Notch signaling has also been reported to negatively regulate TLR-triggered inflammatory responses through ERK inactivation (46). Thus, the association between Notch signaling and inflammation remains debated and unclear.

Based on the findings highlighted earlier and our previous studies on sepsis, we tried to elucidate both the role of Notch signaling during LPS-induced inflammation and the interaction of SIRT1 with Notch signaling. In this experiment, we used LPS-stimulated macrophages as an inflammation cell model and LPS-injected mice as an animal model. First, we confirmed that LPS treatment led to marked inflammation and aggravated organ injury in mice: LPS injection resulted in an increase in pro-inflammatory cytokines within a brief time, and during this period, SIRT1 expression was repressed (Figure 1). Notably, when we activated SIRT1 in macrophages by using its selective activator SRT1720 (39), the expression of pro-inflammatory cytokines was decreased, and this was accompanied by a reduction in LPS-induced mortality in mice injected with SRT1720 (Figure 2A; Figure S1 in Supplementary Material). Conversely, transfection of SIRT1 siRNA into cells led to an increase in the levels of pro-inflammatory cytokines after LPS stimulation (Figure 2B). We next used a transgenic animal model to further verify the observed effects *in vivo*. Following LPS injection at the same dose, more severe organ injury was detected in *sirt1*^−/−^ mice than in WT mice (Figure 3A), and the expression of pro-inflammatory cytokines was also increased in *sirt1*^−/−^ mice (Figure 3B). Accordingly, after LPS stimulation, *sirt1*^−/−^ macrophages expressed markedly increased levels of pro-inflammatory cytokines as compared with macrophages from WT mice (Figure 4). Finally, when mice were treated with the SIRT1 activator SRT1720, LPS-induced organ injury was alleviated, and the survival rate of mice exposed to LPS was increased (Figures S1 and S2 in Supplementary Material). Thus, in inflammation and sepsis, the SIRT1 level was decreased and this led to severe damage, whereas SIRT1 activation protected against inflammation and sepsis.

As mentioned in Section “Introduction,” Notch signaling plays a key role in inflammation, and SIRT1 is reported to inhibit Notch-mediated transcription. Therefore, we investigated whether SIRT1 was associated with Notch signaling in inflammation. When RAW264.7 macrophages were stimulated with LPS, the transcription of Notch1, Notch2, and Hes1 was increased significantly (Figure 5A), and co-IP results showed that SIRT1 and NICD interacted directly in these cells (Figure 5B), as much as in the endothelium (47). Moreover, when SIRT1 was depleted in macrophages either through siRNA transfection or gene knockout, the levels of Hes1 and NICD were increased considerably. Following LPS stimulation, NICD levels in *sirt1*^−/−^ macrophages and SIRT1-siRNA-transfected macrophages were higher than those in control cells only treated with LPS (Figures 6A,B). Thus, SIRT1 inhibition appeared to lead to the activation of Notch signaling. In LPS-induced macrophages, NICD acetylation was increased (Figure 6C). In the absence of LPS stimulation, addition of MG132, a proteasome inhibitor, resulted in an increase in the NICD level, which indicated that the instability of NICD was at least partly responsible for its low level in macrophages. When macrophages were transfected with SIRT1 siRNA and treated with MG132, NICD acetylation was increased substantially (Figures 6D,E). Thus, to examine the effect of SIRT1 activity, we expressed a dominant-negative SIRT1 mutant (H363Y) plasmid or WT SIRT1 in macrophages and stimulated the cells with LPS. Our results showed that the NICD acetylation level after LPS stimulation was higher in cells overexpressing mutant SIRT1 than in cells expressing WT SIRT1, and, accordingly, NICD acetylation after LPS treatment was higher in *sirt1*^−/−^ macrophages than in WT macrophages (Figures 6F,G; Figure S4 in Supplementary Material). These results demonstrated that SIRT1 could negatively regulate the activation of Notch signaling in inflammation.

We next addressed the key question of what changes occur in inflammation in macrophages and animals when the activation of Notch signaling is regulated. For this purpose, we used *RBP-J*^−/−^ macrophages, because *RBP-J* is a critical DNA-binding transcription factor associated with Notch signaling in mouse. After LPS stimulation, the levels of pro-inflammatory cytokines in *RBP-J*^−/−^ macrophages were markedly lower than those in macrophages from WT mice (Figure 7A). The levels of pro-inflammatory cytokines in macrophages were also decreased after treatment with DAPT, an inhibitor of Notch signaling (Figure S3 in Supplementary Material). Moreover, when the same dose of LPS was administered to *RBP-J*^−/−^ mice and their WT littermates, the mortality of *RBP-J*^−/−^ mice was found to be lower than that of the WT mice (Figure 8A), and, furthermore, organ injury was milder and the levels of pro-inflammatory cytokines were lower in *RBP-J*^−/−^ mice than in WT mice (Figures 8B–D).

NF-κB is part of a critical protein complex that participates in immunity and inflammation and in cell proliferation, differentiation, and survival (48). However, the relationship between Notch signaling and the NF-κB pathway remains debated (46, 49, 50). Notch1 signaling was reported to enhance NF-κB activity in macrophages after stimulation of the cells with LPS. Moreover, basal NF-κB activity in RAW264.7 cells transfected with an NICD plasmid appeared to be slightly elevated, and pretreatment of RAW264.7 cells with DAPT diminished NF-κB activity in RAW264.7 cells (51). Given these findings, we examined NF-κB activation in macrophages. Our results showed that *RBP-J* knockout in macrophages led to reduced phosphorylation of p65 and IκBα (Figure 7B), which indicated that inhibition of Notch signaling resulted in the inactivation of the NF-κB pathway.

**Figure 9:**
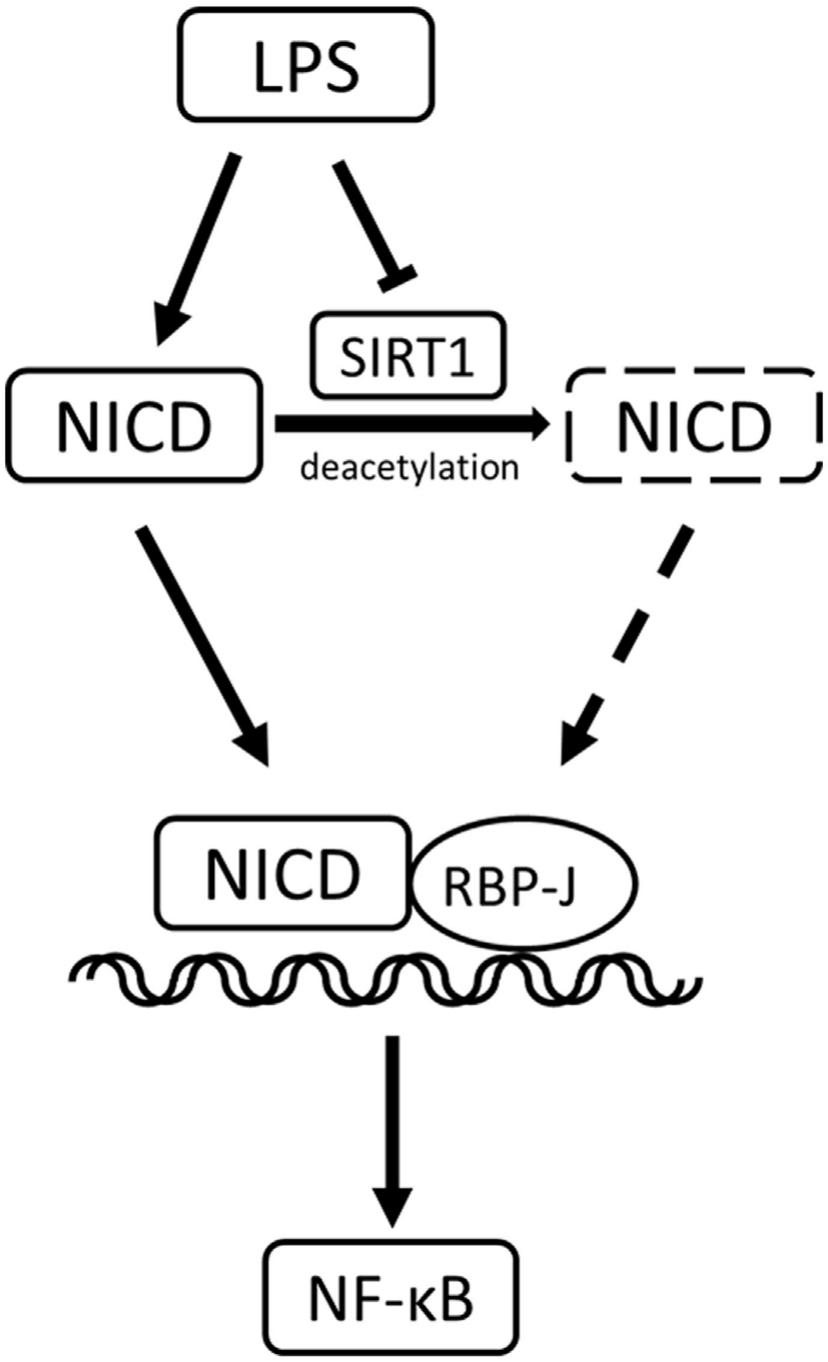
Sirt1-Notch interaction in lipopolysaccharide (LPS)-induced macrophages. In LPS-induced inflammation, SIRT1 was suppressed, and Notch signaling was activated. SIRT1 caused the deacetylation of intracellular domain of Notch (NICD), which led to NICD degradation and inhibition of Notch signaling. The inactivation of Notch signaling reduced the activation of the NF-κB pathway in inflammation.

In conclusion, our study yielded these key results (Figure 9): in LPS-induced inflammation, SIRT1 was downregulated, and Notch activation was increased. SIRT1-mediated deacetylation of the NICD led to NICD-degradation and Notch signaling inhibition. The inactivation of Notch signaling, in turn, resulted in a decrease in the activation of the NF-κB pathway and ultimately in a reduction in the expression of pro-inflammatory cytokines and an alleviation of inflammation.

## Ethics Statement

This study was carried out in accordance with the principles of ARRIVE approved by the Ethics Committee of Xijing Hospital, affiliated with the Fourth Military Medical University (No: XJYYLL-2015206).

## Author Contributions

XB, TH, and YL contributes equally as first author. XB and DH designed the project. XB, TH, and YL performed some of the experiments, analyzed data, and wrote the manuscript. JZ, XL, and KW performed some of the experiments and analyzed data. FH performed part of the data analyses and helped revised the manuscript. JS, WZ, YZ, and WC contributed reagents, materials, and analysis tools. XB, TH, and DH revised and approved the final submission.

## Conflict of Interest Statement

The authors declare that the research was conducted in the absence of any commercial or financial relationships that could be construed as a potential conflict of interest. The reviewer TB and handling Editor declared their shared affiliation.

## Supplementary Material

Figure S1 SRT1720 injection increased the survival rate of mice and decreased the levels of pro-inflammatory cytokines after lipopolysaccharide (LPS) exposure. **(A)** Mice were injected with SRT1720 or saline and then injected with LPS. The survival rate was calculated over 72 h. **(B)** The levels of IL-1β, IL-6, and TNF-α in blood were assessed using commercial ELISA kits. ***p* < 0.01 compared with mice injected with PBS; *n* = 6.

Figure S2 SRT1720 injection potently alleviated lipopolysaccharide (LPS)-induced organ injury in mice. **(A)** Hematoxylin and eosin staining of heart, liver, lung, and kidney tissues from SRT1720-injected and control (saline-injected) mice exposed to LPS; sections were examined and photographed under a microscope. **(B)** Blood from the left ventricle was collected and the levels of creatinine (Cr), blood urea nitrogen (BUN), alanine aminotransferase (ALT), and aspartate transaminase (AST) were assessed using commercial ELISA kits. ***p* < 0.01 compared with LPS-treated control mice; *n* = 6. Scale bar = 50 µm.

Figure S3 DAPT treatment lowered the levels of pro-inflammatory cytokines in lipopolysaccharide (LPS)-stimulated macrophages. Peritoneal macrophages collected from C57BL/6 mice were exposed to PBS (control), DAPT, LPS, or LPS + DAPT, and then the mRNA levels of IL-1β, IL-6, TNF-α, and Hes1 were determined using RT-PCR. ***p* < 0.01 compared with the LPS group; *n* = 6.

Figure S4 Quantification of the results in Figure 6F, showing higher intracellular domain of Notch acetylation after transfection of H363Y SIRT1 plasmid than wild-type SIRT1 plasmid. Data were analyzed using Graph Pad Prism 5.01 software. ***p* < 0.01.
