# Supplementary figures and images for: Acetylation-Dependent Regulation of Notch Signaling in Macrophages by SIRT1 Affects Sepsis Development
Source: Front Immunol. 2018 May 7;9:762. doi: 10.3389/fimmu.2018.00762 (PMC5949384; doi:10.3389/fimmu.2018.00762)

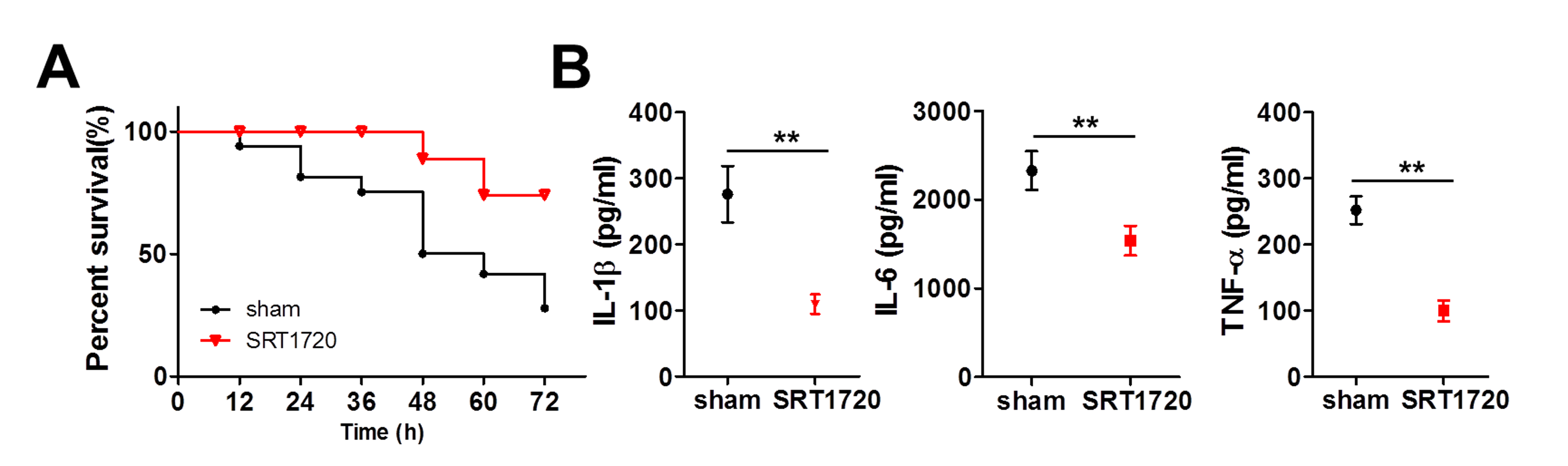

Supplement: Figure S1 — SRT1720 injection increased the survival rate of mice and decreased the levels of pro-inflammatory cytokines after lipopolysaccharide (LPS) exposure. (A) Mice were injected with SRT1720 or saline and then injected with LPS. The survival rate was calculated over 72 h. (B) The levels of IL-1β, IL-6, and TNF-α in blood were assessed using commercial ELISA kits. **p < 0.01 compared with mice injected with PBS; n = 6. [file Image_1.TIF]

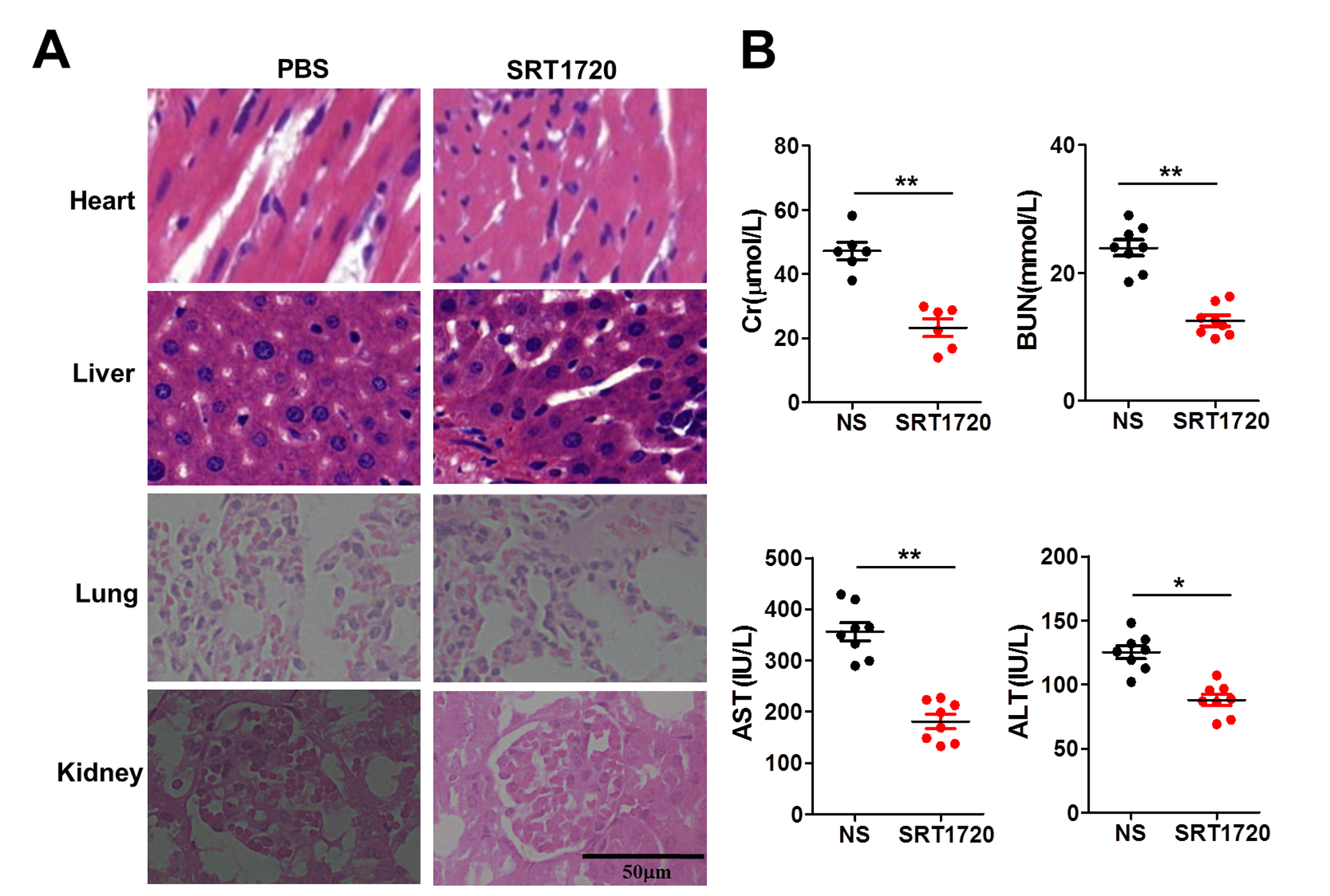

Supplement: Figure S2 — SRT1720 injection potently alleviated lipopolysaccharide (LPS)-induced organ injury in mice. (A) Hematoxylin and eosin staining of heart, liver, lung, and kidney tissues from SRT1720-injected and control (saline-injected) mice exposed to LPS; sections were examined and photographed under a microscope. (B) Blood from the left ventricle was collected and the levels of creatinine (Cr), blood urea nitrogen (BUN), alanine aminotransferase (ALT), and aspartate transaminase (AST) were assessed using commercial ELISA kits. **p < 0.01 compared with LPS-treated control mice; n = 6. Scale bar = 50 µm. [file Image_2.TIF]

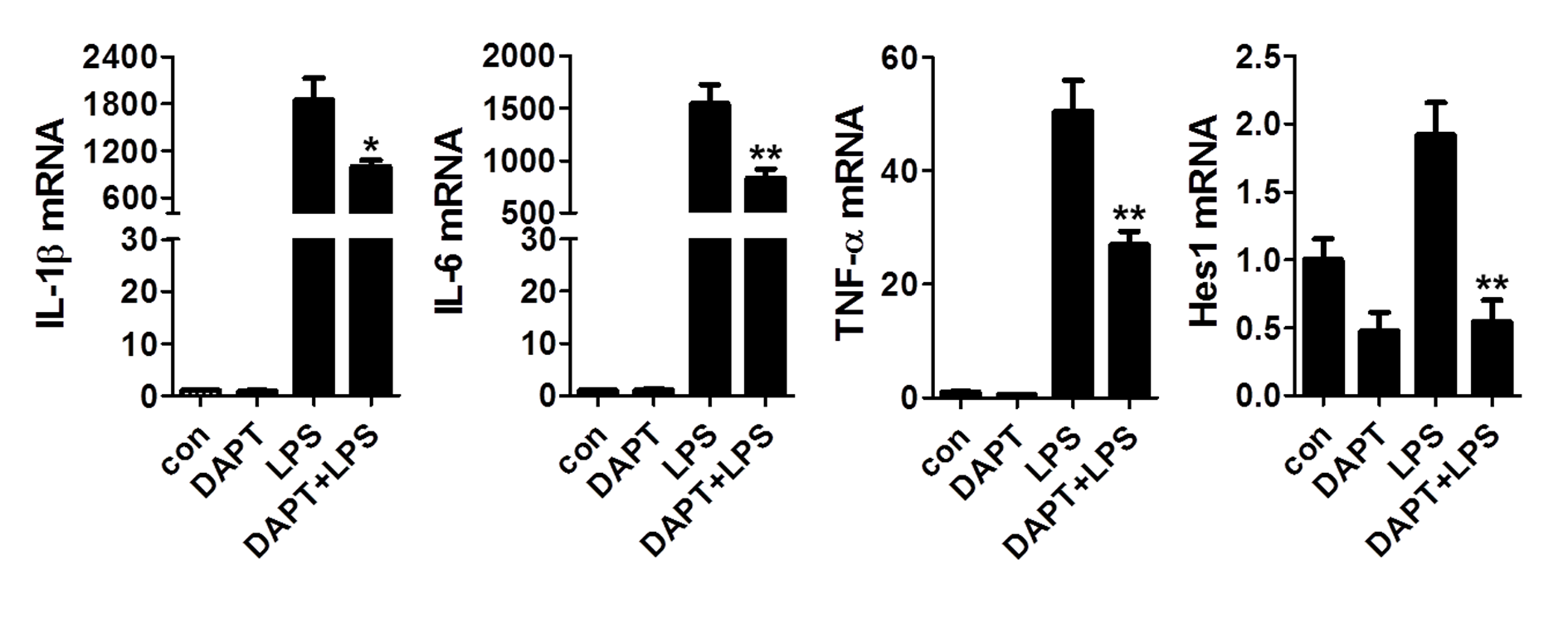

Supplement: Figure S3 — DAPT treatment lowered the levels of pro-inflammatory cytokines in lipopolysaccharide (LPS)-stimulated macrophages. Peritoneal macrophages collected from C57BL/6 mice were exposed to PBS (control), DAPT, LPS, or LPS + DAPT, and then the mRNA levels of IL-1β, IL-6, TNF-α, and Hes1 were determined using RT-PCR. **p < 0.01 compared with the LPS group; n = 6. [file Image_3.TIF]

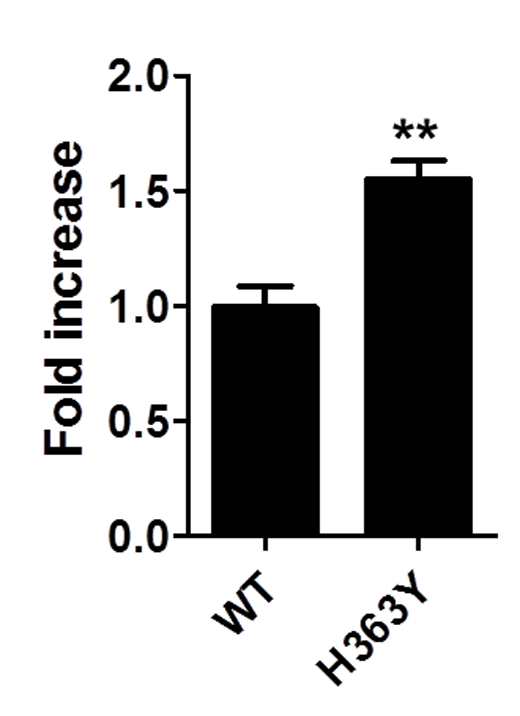

Supplement: Figure S4 — Quantification of the results in Figure 6F, showing higher intracellular domain of Notch acetylation after transfection of H363Y SIRT1 plasmid than wild-type SIRT1 plasmid. Data were analyzed using Graph Pad Prism 5.01 software. **p < 0.01. [file Image_4.TIF]
